# Population-based study of preventable infections in hospitalized patients with systemic lupus erythematosus

**DOI:** 10.1186/ar3948

**Published:** 2012-09-27

**Authors:** CE Barber, C Barnabe, D Marshall, J Esdaile

**Affiliations:** 1 University of Calgary, AB, Canada; 2 Arthritis Research Center of Canada, Vancouver, BC, Canada

## Background

Infection is a prominent cause of morbidity and mortality in patients with systemic lupus erythematosus (SLE). The proportion of hospitalizations in patients with SLE due to preventable infections is unknown. The objectives of this study were to: determine the proportion of hospitalizations in SLE patients due to preventable infections; and determine whether preventable infections are associated with lengths of stay ≥14 days or mortality.

## Methods

This study was a retrospective cohort study using a provincial administrative dataset of hospitalizations (Discharge Abstract Database (DAD)) in Alberta, Canada. A total of 1,626 SLE patients with hospitalizations during fiscal years 2002 to 2009 were identified from the DAD using the International Classification of Diseases-10-CA (ICD-10-CA) code M32.x. Outcome measures of preventable infections (influenza, *Streptococcal pneumonia*, tuberculosis and unspecified pneumonia) were identified during readmissions in 2009/10 using ICD-10 codes. Comparisons between admissions with and without infections were computed using admission and patient characteristics: Student's *t *test and the Wilcoxon rank-sum tests were used for continuous variables and the chi-square test was used for categorical variables. Generalized estimating equation models were constructed to examine associations between infections and the outcomes of long length of stay (dichotomized to ≥14 or <14 days) and mortality.

## Results

In total, 312 SLE patients had 504 admissions in 2009/10. Preventable infections accounted for 9.3% of all admissions. Patients with infections had similar demographic characteristics and co-morbidities compared with those without infections with the exception of chronic pulmonary diseases that were more common in those with infections (41.4% vs. 18.3%, *P *= 0.003). Hospitalizations with preventable infections were associated with longer median length of stay (7.5, IQR 4.4 to 23.6 days vs. 4.7, IQR 2.3 to 11.0 days, *P *< 0.001) and higher mortality rates (12.8% vs. 5.5%, *P *= 0.047). Generalized estimated equations revealed that infections were associated with length of stay ≥14 days (OR = 2.59, 95% CI = 1.31 to 5.11, *P *= 0.006) but not mortality (OR = 2.13, 95% CI = 0.81 to 5.62, *P *= 0.128).

## Conclusion

Nearly one in 10 admissions in SLE patients previously hospitalized are due to or complicated by preventable infections. The preventable infections are associated with an increased length of stay and therefore greater resource utilization. These results highlight the importance of preventative vaccinations in SLE patients. Further studies are warranted to determine where gaps in vaccination and infection prevention occur in this population.

